# Chronic Granulomatous Disease: A Multicentre Study of DHR Flow Cytometry Results and Clinical Correlations in Malaysia

**DOI:** 10.21315/mjms-03-2025-149

**Published:** 2025-08-30

**Authors:** Nahla Mohammed Saif, Zarina Thasneem Zainudeen, Intan Juliana Abd Hamid, Nurul Khaiza Yahya

**Affiliations:** 1 Primary Immunodeficiency Diseases Group, Department of Clinical Medicine, Advanced Medical and Dental Institute, Universiti Sains Malaysia, Pulau Pinang, Malaysia; 2 Immunology Department, School of Medical Sciences, Universiti Sains Malaysia, Health Campus, Kelantan, Malaysia

**Keywords:** chronic granulomatous disease, dihydrorhodamine assay, cells percentages, mean fluorescence intensity (MFI), internal reference range

## Abstract

**Background:**

Chronic granulomatous disease (CGD) is an inherited primary immunodeficiency disease that results from a defect in one of the respiratory burst oxidases (NADPH oxidase) genes that form its components. The diagnostic laboratory assessment for CGD includes evaluation of NADPH oxidase function in neutrophils, using DHR test. The main objective of this project is to develop an internal reference range by determining the relative proportion of oxidising cells percentages and the mean fluorescence intensity (MFI) for complete interpretation of the test results and more accurate results.

**Methods:**

A retrospective analysis was conducted on 107 individuals referred for DHR testing from Institut Perubatan dan Pergigian Termaju (IPPT) and Hospital Universiti Sains Malaysia (HUSM). Purposive sampling was employed. Logistic regression was used to explore relationships between groups and test parameters, and receiver operating characteristic curve (ROC) analysis assessed test precision.

**Results:**

From our findings, the optimum cut-point for IPPT to differentiate CGD patients from healthy group with fMLP% was 0.26%, and FMLP MFI was 9.15, PMA% was 58.70%, and PMA MFI was 50.00. For the HUSM data, the best possible cut-point for fMLP% was 3.86%, FMLP MFI was 999.50, PMA% was 45.69%, and PMA MFI was 1130.50. The parameters showed a good analytical ability since all the area under the curve (AUC) values were significant (*P* > 0.5).

**Conclusion:**

This study confirmed the importance of developing an internal reference range for accurate diagnosis of CGD. This data showed a difference between two centres in terms of test results and cut-points, highlighting the need for standardised reference ranges in diagnostic testing.

## Introduction

Chronic granulomatous disease (CGD) is a genetically heterogeneous disorder resulting from a mutation in any one of the five genes responsible for the formation of the nicotinamide adenine dinucleotide phosphate (NADPH) oxidase components of phagocytes, which leads to primary immunodeficiency (1). CGD is classified based on the type of NADPH mutation into X-linked CGD, which is caused by a mutation in the CYBB gene, which encodes for gp91phox. In addition, there is autosomal recessive (AR) CGD which is caused by a mutation in one of these genes: NCF4, CYBA, NCF2, and NCF1, which encodes for these protein complexes: p40phox, p22phox, p67phox, and p47phox, respectively (2). All these protein complexes are important for superoxide generation. Therefore, having this mutation can cause the loss of H_2_O_2_ production and affect the mechanism of neutrophil killing in CGD (3).

The danger of this disease is that it cripples the first line of defence in the human immune system, which is the innate immunity (4). Annually, the acute infection rate is approximately 0.3 per patient, where the most affected organ by CGD is the lung (around 40% to 85% of patients) (5). CGD patients have other manifestations, including photosensitivity, eczema, lupus-like rash, as well as malar rash (6). Other symptoms mainly include pneumonia due to *Staphylococcus aureus*, *Burkholderia cepacia*, and Gram-negative bacteria (7).

At present, the curative treatment method for CGD is haematopoietic stem cell transplantation (HSCT) (8) from a human leukocyte antigen (HLA)-matching related donor (MRD) (9), which might invert the inflammatory and infective diseases (10). The total survival rate of CGD after HSCT is about 90% until adulthood age, while some have reported that the percentage of survival among CGD is around 81%. However, in developing countries, the rate is lower than that (10, 11).

The CGD diagnosis is based on the absence of the respiratory burst in phagocytes. When phagocytes of healthy individuals are stimulated, they will produce superoxide, but CGD patients’ cells will not produce superoxide after stimulation, whereas some of the X-linked carriers’ cells will produce it. The diagnosis is done by using either the dihydrorhodamine (DHR) test or the nitroblue tetrazolium (NBT) test (12). A DHR flow cytometry test is used to quantify NADPH oxidase activity, which helps to determine reactive oxygen intermediates (ROI). The diagnostic laboratory assessment for CGD includes evaluation of NADPH oxidase function in neutrophils, using the more analytically sensitive DHR test.

An internal reference range is an essential and helpful element that will aid in determining CGD (13). The obtained results will be compared to the reference range to identify the individuals as patients, carriers, or healthy correctly, and to evaluate the physiological functions. Therefore, it will aid in early detection, determining the appropriate treatment, and monitoring the cases. Furthermore, the DHR test and corresponding reference range will have both the proportion of neutrophil cells (%) and the mean fluorescence intensity (MFI) of the cells for full interpretation and correct diagnosis of CGD (14). The MFI value corresponds to the quantity of the antibodies that are bound to the neutrophils’ surface antigens (NADPH oxidase) on the plasma membrane of phagocytes. So, the exact numbers of the antigens will be quantified in each cell.

Due to the lack of a normal reference range of the DHR test in Malaysia, the study will be improved by creating internal references that are more accurate, assisting with disease diagnosis and management. The main objective of this study is to develop an internal reference range by determining the relative proportion of oxidising cells percentages and MFI, which will provide a complete interpretation of the test results and make the results more accurate and reliable. This internal reference range will also help in avoiding false-negative results.

## Methods

In this retrospective study, all available and completed files (file format:.fsc) that consist of all the subgroups (unstimulated, Escherichia coli, fMLP, and PMA) were retrieved from the Institut Perubatan dan Pergigian Termaju (IPPT) and Immunology Laboratory, Hospital Universiti Sains Malaysia (HUSM) between January 2010 and December 2020. The sample size was calculated using the single mean formula (with a 20% dropout), and purposive sampling was performed. The inclusion criteria were all subjects with DHR results and all available results were in.fcs files. Those with incomplete results or subjects with secondary immunodeficiency were excluded from the study.

The available and complete flow data were analysed using a flow cytometry data analysis software called FlowJo. In FlowJo, each DHR file consists of control and patient data. The clinical information (e.g. presenting feature, causative microorganisms, and diagnosis), immunological results (i.e. complete blood count (CBC), IgG, IgE, IgA, IgM, and CD3, 4, 8, 19 markers), socio-demographics (e.g. age, sex, and ethnicity), and the clinical diagnosis were collected and analysed to determine the type of infection and their relations to the DHR+ results. The study was approved by the JEPeM USM Code: USM (HREC) (21010042).

### Statistical Analysis

The mean and percentage of the oxidising cells of all subjects were retrieved from the statistics analysis. The histogram tested the normality of data distribution. The *t*-test was used to compare the significant differences between two groups with normal distributed values, while the Mann-Whitney U test was performed for data with a non-normal distribution. The descriptive statistic was used to get the mean and the standard deviation for all study groups. The association between the dependent variables (healthy, carriers, and patients) and the independent variables (fMLP%, MFI fMLP, PMA%, and MFI PMA) was assessed using logistic regression analysis. Finally, the cut-off point of the parameter was determined by a receiver operating characteristic curve (ROC). Also, the area under the curve (AUC) was used to determine the ability of the DHR test to differentiate between the disease and non-disease individuals.

## Results

### Demographic of Sample Cohort from IPPT and HUSM

Data from 107 individuals from IPPT and HUSM were analysed. The mean age for normal individuals was 6.40 years (SD = 4.91), for carriers was 19.00 years (SD = 8.24), and for patients was 9.50 years (SD = 12.02). Gender information was available for 47.37% of individuals from the HUSM Immunology Laboratory, comprising three males (15.79%) and six females (31.58%). Age data were available for 10.53% of these individuals, with a mean age of 5 years (SD = 2.82). Among the remaining 88 individuals from IPPT, 41 were males (46.59%) and 35 were females (39.77%). Age information was available for 21.59% of the IPPT samples, with a mean age of 9 years (SD = 9.42) (Table 1).

All identified carriers in our cohort were female, and two distinct peaks were observed in the DHR assay histograms following PMA stimulation, consistent with patterns seen in heterozygous carriers of X-linked CGD. The mean PMA% in the three IPPT patients was 15.54 (SD = 26.27), while the single HUSM patient exhibited a PMA% of 0.09% following stimulation (data not shown).

### Infectious Complications and Isolated Microorganisms from IPPT Cohort

Predominant clinical manifestations from the IPPT cohort are listed in Table 2. For individuals with abnormal DHR results, mumps infection, episodes of urinary tract infection, left pleural collection secondary to abscess, sepsis, and fever were the commonest clinical presentations. As for individuals with normal DHR results, there were several common infections and complications, including fever, fit, vomiting, diarrhoea, *Klebsiella pneumoniae* sepsis, upper respiratory tract infection (URTI), and left ear otitis media, to name a few. The most common clinical presentations in individuals with positive and negative DHR tests were fever and sepsis.

Overall, there were 12 types of pathogens isolated from patients with normal and abnormal DHR tests as shown in Table 3. The most common isolated organisms from patients’ culture are *Aspergillus fumigatus*, *E. coli*, *K. pneumoniae*, and *Proteus mirabilis*. While the most common isolated organisms from non-CGD patients’ culture are *Burkholderia pseudomallei*, *Salmonella*, *Elizabethkingia meningoseptica*, HHV-6, Dengue virus, influenza A, and adenovirus. *K. pneumoniae* was found in both groups. An infectious history was not established for the HUSM cohort due to limited information.

### Quantification of DHR Parameter Values of The Respiratory Burst Activity in Neutrophils (Healthy, Carriers, and Patients)

Out of 88 individuals from the IPPT (77 healthy, eight carriers, and three patients) and 19 from HUSM (18 healthy and one patient), a descriptive analysis indicated that healthy individuals consistently exhibited higher mean neutrophil respiratory burst activity across all parameters. Statistically significant differences were observed within the IPPT cohort for *E. coli* % (*P* = 0.05), *E. coli* MFI (*P* = 0.02), fMLP% (*P* = 0.00), PMA% (*P* = 0.00), and PMA MFI (*P* = 0.01), highlighting clear distinctions between healthy individuals, carriers, and CGD patients (Supplementary 1). In contrast, although HUSM data showed similar trends of reduced activity in the patient, no statistically significant differences were detected between groups (data not shown).

### Association of DHR Parameters According to Centres and Status of Samples (Normal vs Abnormal)

Logistic regression analysis further supported these findings. In the IPPT cohort, the patient status was negatively associated with neutrophil respiratory burst activity, shown by fMLP% (coefficient = −9.62; OR = 0.00; 95% CI: 0.00, 75.42), fMLP MFI (coefficient = −0.04; OR = 0.95; 95% CI: 0.84, 1.08), PMA% (coefficient = −1.25; OR = 0.29; 95% CI: 0.00, 2.42), and PMA MFI (coefficient = −0.07; OR = 0.93; 95% CI: 0.85, 1.01), indicating reduced activity in the patients (Supplementary 2). In the HUSM cohort, fMLP% showed a weak positive trend (coefficient = 0.15; OR = 1.16; 95% CI: 0.70, 1.92), while both fMLP MFI (coefficient = −0.01; OR = 0.99; 95% CI: 0.98, 1.00) and PMA MFI (coefficient = −0.04; OR = 0.96; 95% CI: 0.01, 554.38) were negatively associated, indicating lower fluorescence intensity in the patients. PMA% data were not available for HUSM (Supplementary 3). Overall, these results emphasise that CGD patients generally display lower oxidative activity, particularly in the MFI values.

### Determining a Cut-point Value of DHR Test Parameters by the ROC for IPPT and HUSM

A cut-point value of DHR test parameters was determined by ROC for IPPT and HUSM. The analysis was done separately for the IPPT and the HUSM data sets. The ROC analysis displayed that all the parameters of both centres showed a good analytical ability, as all the AUC values were significant (*P* > 0.5). As tabulated in Table 4, the cut-point of fMLP%, fMLP MFI, PMA%, and PMA MFI were 0.26%, 9.15, 58.70%, and 50.00, respectively. The PMA% had the highest sensitivity of 1.00, which indicates that this parameter is more efficient in diagnosing CGD. On the other hand, according to HUSM data, the calculated cut-point of fMLP%, fMLP MFI, PMA%, and PMA MFI were 3.86%, 999.50, 45.69%, and 1,130.50, respectively, as shown in Table 5. All the parameters’ cut-points have zero specificity. Hence, these parameters may have the best ability to detect CGD correctly.

## Discussion

In Malaysia, CGD is the third most frequent primary immunodeficiency reported (11). The gold standard for diagnosing CGD is the DHR flow cytometry assay, particularly since genetic testing was not routinely available prior to 2020. Distinguishing CGD patient from myeloperoxidase (MPO) deficiency can be achieved by creating an internal reference range consisting of both percentages and MFI of neutrophils’ respiratory burst activities. To achieve that, we decided to collect data from IPPT and HUSM that contained.fcs files of DHR test results of CGD cases and healthy controls.

### Demographic of Sample Cohort from IPPT and HUSM

This study involved DHR test results files for 107 individuals from IPPT and HUSM, where four of them were patients, 95 were healthy, and eight were carriers with the mean age of 9.50 (SD = 12.02), 6.40 (SD = 4.91), and 19.00 (SD = 18.24) years, respectively. The number of CGD patients in our study was small (*n* = 4), due to the nature of our study, as it was difficult to get all the recorded data. One patient from HUSM had to be excluded for further analysis, as the patient has another underlying disease, which is B-cell lymphoma. About 11 healthy data points from the IPPT had to be excluded because the flow data either contained fewer cells (unable to gate for analysis), were corrupted, or had incomplete information. The mean age of our patients was 9.50 years, which is older than a previous study (15) that involved 117 CGD patients with a mean age of 4.25 years. As discussed previously, CGD symptoms usually manifest during childhood due to recurrent infections by different pathogens such as bacteria, fungi, and viruses, and this is noted in this study.

### Infectious Complications and Isolated Microorganisms from IPPT Cohort

Regarding clinical manifestations, CGD patients from IPPT were presented with mumps infection, episodes of urinary tract infection (UTI), left pleural collection secondary to abscess, sepsis, and fever. In contrast, individuals with normal DHR test had vomiting, diarrhoea, fever with chesty cough, febrile fit with runny nose, bronchopneumonia, left ear otitis media, as well as other infectious diseases. We observed that CGD patients were characterised by left pleural collection secondary to abscess, and mumps infection over those with normal DHR test. Therefore, identifying and understanding these clinical manifestations could help in requesting the DHR test earlier before developing complications. However, we could not proceed with statistical analysis in view of the limited number of patients.

Besides that, severe infections such as sepsis were found in one CGD patient from IPPT. Our findings were contrary to previous findings, where sepsis accounted for 31% in oxidase null patients and 27% in oxidase residual patients (2). This inconsistency might have been caused by the smaller sample size in our study compared to their sample. Recurrent infection caused by *Staphylococcus* species, such as *E. coli* or *Klebsiella*, and saprophytic fungi, especially *Aspergillus* species, affects the majority of CGD patients (16). In respect of infected microorganisms, the most common isolated microorganisms from CGD patients’ culture in this study were *A. fumigatus*, *E. coli*, *K. pneumoniae*, and *P. mirabilis* from those with positive DHR test. However, there were similarities between our findings and previous studies (11, 17–19) in terms of infected microorganism, namely *E. coli, Klebsiella* species, and *Aspergillus* species. Therefore, due to this finding, we concluded that *E. coli, Klebsiella* species, and *Aspergillus* species were the most isolated pathogens in this study. These pathogens require prompt treatment once they are identified to avoid further complications and risk of deterioration.

### Quantification of DHR Parameter Values of The Respiratory Burst Activity in Neutrophils (Healthy, Carriers, and Patients)

Descriptive statistical analysis was used to show the characteristics and the normal distribution of parameters’ values (unstimulated %, unstimulated MFI, *E. coli* %, *E. coli* MFI, fMLP%, fMLP MFI, PMA%, and PMA MFI) for each centre separately. The reason for doing the test separately was that when the mean and standard deviation for all centres were tested, it was found that there was a statistically significant difference between healthy, carriers, and patients, only in two parameters, which were *E. coli* % and PMA%. This finding was in line with the proposed hypothesis of this study, which suggested that each centre needs to have its internal reference range due to the variations in the percentage and the MFI values of respiratory burst activity, as seen in this study, where MFI values from the HUSM cohort were bigger than those of the IPPT cohort.

For the IPPT groups of healthy, carriers, and patients, there was a statistically significant difference between them found in *E. coli* %, *E. coli*, fMLP%, PMA%, and PMA MFI parameters. The mean of the parameter values of healthy was the highest, followed by carriers and patients. This finding was expected and indicated that healthy individuals have normal neutrophil respiratory burst activity after stimulation with different stimuli of *E. coli*, fMLP, and PMA. Meanwhile, patients have low neutrophil respiratory burst activity due to a defect in their respiratory burst activities. The findings in this study were consistent with previous reported findings (20), which calculated the stimulation index (SI) of PMA MFI for healthy, XL-CGD patients, and AR-CGD patients, which were 193.5, 1.23, and 23.7, respectively. Although our method of calculating the mean of PMA MFI values was different, similar observations were obtained, where the neutrophil activity values were high in the healthy group and low in CGD patients. We choose to represent our data in MFI as it gives a true reflection of all the neutrophil respiratory burst activity after stimulation. SI can be easily derived by dividing the stimulated and the unstimulated MFI (for each stimulant) (20). Both SI and MFI values have been accepted in most diagnostic laboratories.

On the other hand, the HUSM data showed no statistically significant difference between the healthy and patient groups. This observation was due to the limited amount of data from HUSM. We postulated that the results may be different if more data on patients were available.

### Association of DHR Parameters According to Centres and Status of Samples (Normal vs Abnormal)

Binary logistic regression analysis of IPPT parameters revealed that lower values of fMLP%, fMLP MFI, PMA%, and PMA MFI were associated with CGD, while higher values corresponded to healthy individuals. Although these associations aligned with expected trends, they did not reach statistical significance. Similarly, analysis of HUSM data showed that low values of fMLP%, fMLP MFI, and PMA MFI were linked with CGD, while high values were associated with healthy individuals. PMA% values were not available for HUSM during the analysis. Due to the limited availability of comparable studies, direct comparisons with existing literature were not possible.

Despite the lack of statistical significance, the observed relationships suggest that these DHR test parameters are capable of reflecting neutrophil respiratory burst activity and distinguishing between CGD patients and healthy individuals. This observation supports the potential utility of these parameters in diagnostic interpretation and reinforces their role in determining effective cut-off points for more accurate diagnosis.

### Determining a Cut-Point Value of DHR Test Parameters by The ROC for IPPT and HUSM

The ROC curve analysis is a graphic representation of the connection between sensitivity and specificity that aids in the selection of the appropriate cut-point by calculating the results of the diagnostic test. In clinical practice, ROC curves are used to determine the best cut-points for a test, where the best cut-point has the lowest false positive value and the highest true positive value (21).

According to the ROC analysis, the optimum cut-point for IPPT to differentiate CGD patients from the healthy group, with fMLP% was 0.26%, and FMLP MFI was 9.15. While the PMA% cut-point was 58.70% and the PMA MFI cut-point was 50.00. The parameters have a good predictive ability to differentiate between non-diseased and diseased, since all the parameters had the ROC of more than 0.05. On the other hand, according to the HUSM data, the best possible cut-point for fMLP% was 3.86%, and FMLP MFI was 999.50. Whilst the PMA% cut-point was 45.69% and PMA MFI was 1130.50. Moreover, the AUC of the parameters was also high. Our findings differed from those of the Mayo Clinic (22), as their reference range values were higher than ours. The Mayo Clinic’s values were generally higher for PMA, PMA MFI, fMLP, and FMLP MFI, while our IPPT cut-off values were lower. The difference in the reference range is due to the bigger sample size included in the Mayo Clinic study.

Another study (23) used a different method in determining the reference range as an interval. The sample in the study was classified into ten intervals or sub-groups of healthy children, with the first group of individuals aged between zero and one month, and the last group consisting of individuals between the ages of 12 and 18 years old. The reference ranges for each group were determined by calculating the SI (PMA MFI/ unstimulated MFI). The study concluded that due to the passage of the mother’s neutrophils to the neonate, there was no difference in the SI values between the other age groups with the newborn. In terms of difference, their method was different compared to our method used where we determined the cut-points for each parameter separately (fMLP%, fMLP MFI, PMA%, and PMA MFI) by ROC. Our study aimed to get the exact value of neutrophil activity specifically, but the intervals method does not give us what we were aiming to achieve since it does not provide a certain value for all the parameters.

The sensitivity and specificity of the DHR test were 100%, with 100% positive predictive value (PPV) and 100% negative predictive value (NPV) (data not shown). However, the DHR test is very efficient in identifying CGD-positive cases since 100% of all of the CGD-positive patients who were identified by this test indeed had the disease. A previous study (24) obtained similar sensitivity, PPV, and NPV but slightly lower specificity, which was 98% for the Neutrophil Extracellular Traps (NETs) assay. Hence, we concluded that the DHR test was useful as a diagnostic test for CGD.

However, in our current analysis, there were a few limitations. First, only the percentage of oxidase-positive neutrophils and the MFI of stimulated neutrophils were available, as the raw data files were not accessible during data collation. Moving forward, incorporating parameters such as the SI, neutrophil oxidative index (NOI), and delta MFI would allow for a more comprehensive assessment of the neutrophil oxidative burst, especially when using both PMA and fMLP stimulation.

Additional limitations should also be acknowledged. The primary challenge was the difficulty in retrieving data from both centres due to the Malaysian Movement Control Order (MCO), which restricted access to laboratories and campuses. Consequently, demographic and clinical data for many study participants were unavailable. This situation made it difficult to identify several infectious diseases and isolated microorganisms. Another limitation was our inability to collect the immunological results (CBC, IgG, IgE, IgA, IgM, and CD 3, 4, 8,19 markers) due to the limited data available. In addition, some of the obtained files were incomplete and excluded from the analysis. All these factors made the sample size of the groups (healthy, carriers, patients) unequal, affecting the significant differences between them. Results from this study were carefully interpreted within this limitation and avoided overgeneralising the conclusions.

## Conclusion

In conclusion, this data showed that there was a difference between the two centres in terms of test results and cut-points. This situation was because the assay is user-dependent and each centre has its specific protocol, resulting in different readings and interpretations of the results. We have suggested two cut-points based on two specific centres to be used in their respective laboratory as a reference range. Further work is required, and it is recommended that both centres revisit and revise their reference range by conducting the same method used in this report, but using a larger sample size in the future to get more accurate cut-points.

## Supplementary Material

**Supplementary 1:** The respiratory burst activity in neutrophils from healthy, carrier and CGD patients from IPPT, stimulated with *E. coli*, PMA, or fMLP

	Healthy *N* = 77 Mean (SD)	Patient *N* = 3 Mean (SD)	Carrier *N* = 8 Mean (SD)	*P*-value
Unstimulated oxidizing cells (%)	96.74 (3.90)	99.13 (1.07)	96.03 (3.77)	0.37
Unstimulated MFI	13.78 (63.56)	2.38 (1.49)	3.33 (1.53)	0.33
*E. coli*+oxidizing cells (%)	84.62(19.31)	11.38 (19.15)	57.86 (23.06)	0.05 ^*^
MFI	67.83(42.37)	17.62 (16.71)	57.38 (44.71)	0.02 ^*^
fMLP oxidizing cells (%)	1.39 (1.70)	0.135 (0.07)	1.63 (1.22)	0.00 ^*^
MFI	28.21 (23.23)	19.77 (20.13)	28.81 (22.67)	0.57
PMA oxidizing cells (%)	95.45 (6.82)	15.54 (26.20)	70.63 (7.07)	0.00 ^*^
MFI	154.17(92.86)	19.60 (21.57)	104.68 (91.28)	0.01 ^*^

IPPT = Institut Perubatan dan Pergigian Termaju; fMLP = N-formylmethionine-leucyl-phenylalanine; MFI = mean fluorescent intensity; PMA = phorbol myristate acetate

**Supplementary 2:** Logistic regression analysis result of DHR test parameters from IPPT

Variable	Coefficient	Odds ratio	Standard error	*P*-value	95% confidence interval	

					Lower	Upper
fMLP oxidizing cells %	−9.62	0.00	7.11	0.17	0.00	75.42
Constant	−0.48	0.61	1.19	0.68		
fMLP MFI	−0.04	0.95	0.06	0.49	0.84	1.08
Constant	−2.25	0.10	1.39	0.10		
PMA oxidizing cells %	−1.25	0.29	167.91	0.99	0.00	2.42
Constant	73.75	1.70	10,895.06	0.99		
PMA MFI	−0.07	0.93	0.04	0.09	0.85	1.01
Constant	0.32	1.38	1.20	0.78		

fMLP = N-formylmethionine-leucyl-phenylalanine; MFI = mean fluorescent intensity; PMA = phorbol myristate acetate

**Supplementary 3:** Logistic regression analysis result of DHR test parameters from HUSM

Variable	Coefficient	Odds ratio	Standard error	*P*-value	95% confidence interval	

					Lower	Upper
fMLP oxidizing cells %	0.15	1.16	0.26	0.56	0.70	1.92
Constant	−3.29	0.04	1.36	0.02		
fMLP MFI	−0.01	0.99	0.01	0.34	0.98	1.00
Constant	1.95	7.01	3.45	0.57		
PMA MFI	−0.04	0.96	3.25	0.99	0.01	554.38
Constant	48.21	8.67	3,988.74	0.99		

DHR = dihydrorhodamine; HUSM= Hopsital Universiti Sains Malaysia; fMLP = N-formylmethionine-leucyl-phenylalanine; MFI = mean fluorescent intensity; PMA = phorbol myristate acetate

## Figures and Tables

**Table 1:** Demographic characteristics of all the individuals analysed from the Immunology Department, HUSM and IPPT (*N* = 107)

	Immunology laboratory HUSM	IPPT	All	Age (years) (mean [SD])
Normal	18	77^a^	95	6.40 (4.91)
Carrier	0	8	8	19.00 (18.24)
Patient	1^b^	3	4	9.50 (12.02)
Exclude	1	11^a^	12	

a Total number of normal controls (*N* = 77) after excluding 11 normal controls;

b Total number of patient (*N* = 1) after excluding one patient;

HUSM = Hopsital Universiti Sains Malaysia; IPPT = Institut Perubatan dan Pergigian Termaju

**Table 2:** Infectious complications in the study population in relation to normal and abnormal DHR results

Normal DHR test	Abnormal DHR test
1. Fit	1. Mumps’ infection
2. Fever	2. Episode of urinary tract infection (UTI)
3. Vomiting and diarrhoea	3. Left pleural collection secondary to abscess
4. Acute encephalopathy with biphasic seizure	4. Sepsis
5. Reduced diffusion (AES.D.) due to HHV Type 6	5. Fever
infection	
6. Dengue encephalitis	
7. Klebsiella pneumonia Sepsis	
8. Liver transaminitis	
9. Sepsis	
10. Bronchopneumonia with parenteral diarrhoea	
11. Fever with chesty cough	
12. Febrile fit with runny nose	
13. Bronchopneumonia	
14. Seizure secondary to bronchopneumonia.	
15. Recurrent presumed upper respiratory tract infection (URTI)	
16. Pneumonia left axillary lymphadenitis	
17. Prepuce infection	
18. Left ear otitis media	

**Table 3:** Microorganism isolated from normal and abnormal DHR test

Normal DHR test	Abnormal DHR test
HHV-6 *Klebsiella pneumoniae* Dengue virus *Burkholderia pseudomallei* *Salmonella* Influenza A Adenovirus *Elizabethkingia meningoseptica*	*Aspergillus fumigatus* *Escherichia coli* *Klebsiella pneumoniae* *Proteus mirabilis*

**Table 4:** The expected results cut-point value of IPPT

	Cut-point	Sensitivity	Specificity	AUC
fMLP%	0.26 %	0.80	0.00	0.91
fMLP MFI	9.15	0.95	0.33	0.67
PMA%	58.70 %	1.00	0.00	1.00
PMA MFI	50.00	0.83	0.00	0.94

IPPT = Institut Perubatan dan Pergigian Termaju; AUC = area under the curve; fMLP = N-formylmethionine-leucyl-phenylalanine; MFI = mean fluorescent intensity; PMA = phorbol myristate acetate

**Table 5:** The expected results cut-point value of HUSM

	Cut-point	Sensitivity	Specificity	AUC
fMLP%	3.86 %	0.17	0.00	0.16
fMLP MFI	999.50	0.83	0.00	0.89
PMA%	45.69 %	1.00	0.00	1.00
PMA MFI	1130.50	1.00	0.00	1.00

HUSM = Hopsital Universiti Sains Malaysia; fMLP% = N-Formylmethionine-leucyl-phenylalanine oxidising cells; fMLP MFI = N-formylmethionine-leucyl-phenylalanine mean fluorescence intensity; PMA% = phorbol myristate acetate oxidising cells; PMA MFI = phorbol myristate acetate mean fluorescence intensity; AUC = area under the curve
